# The functional roles of *IGF-1* variants in the susceptibility and clinical outcomes of mild traumatic brain injury

**DOI:** 10.1186/s12929-019-0587-9

**Published:** 2019-12-02

**Authors:** Yu-Jia Wang, Henry Sung-Ching Wong, Chung-Che Wu, Yung-Hsiao Chiang, Wen-Ta Chiu, Kai-Yun Chen, Wei-Chiao Chang

**Affiliations:** 1 Ph.D. Program for Neural Regenerative Medicine, College of Medical Science and Technology, Taipei Medical University and National Health Research Institutes, Taipei, Taiwan; 2 Department of Clinical Pharmacy, School of Pharmacy, Taipei Medical University, Taipei, Taiwan; 3 Department of Surgery, College of Medicine, Taipei Medical University, Taipei, Taiwan; 4 Department of Neurosurgery, Taipei Medical University Hospital, Taipei, Taiwan; 5 Institute of Injury Prevention and Control, College of Public Health and Nutrition, Taipei Medical University, Taipei, Taiwan; 6 Department of Pharmacy, Wan Fang Hospital, Taipei Medical University, Taipei, Taiwan; 7 Master Program for Clinical Pharmacogenomics and Pharmacoproteomics, School of Pharmacy, Taipei Medical University, Taipei, Taiwan; 8 Department of Medical Research, Shuang Ho Hospital, Taipei Medical University, New Taipei City, Taiwan; 9 Pain Research Center, Wan Fang Hospital, Taipei Medical University, Taipei, Taiwan

**Keywords:** Mild traumatic brain injury, Genetic variants, Insulin-like growth factor 1, Anxiety, Depression, Dizziness, Sleep disorders

## Abstract

**Background:**

Insulin-like growth factor 1 (IGF-1) is an important pleiotropic hormone that exerts neuroprotective and neuroreparative effects after a brain injury. However, the roles of *IGF-1* variants in mild traumatic brain injury (mTBI) are not yet fully understood. This study attempted to elucidate the effects of *IGF-1* variants on the risk and neuropsychiatric outcomes of mTBI.

**Methods:**

Based on 176 recruited mTBI patients and 1517 control subjects from the Taiwan Biobank project, we first compared the genotypic distributions of *IGF-1* variants between the two groups. Then, we analyzed associations of *IGF-1* variants with neuropsychiatric symptoms after mTBI, including anxiety, depression, dizziness, and sleep disturbances. Functional annotation of *IGF-1* variants was also performed through bioinformatics databases.

**Results:**

The minor allele of rs7136446 was over-represented in mTBI patients compared to community-based control subjects. Patients carrying minor alleles of rs7136446 and rs972936 showed more dizziness and multiple neuropsychiatric symptoms after brain injury.

**Conclusions:**

*IGF-1* variants were associated with the risk and neuropsychiatric symptoms of mTBI. The findings highlight the important role of IGF-1 in the susceptibility and clinical outcomes of mTBI.

## Background

Traumatic brain injury (TBI) is a global health issue that has become significantly more prominent over the past two decades, with the prevalence rate increasing by 8.4% between 1990 and 2016 [1]. TBI is defined as a disruption of normal brain function caused by an external mechanical force impacting the head [2]. The leading causes of TBI include falls, being struck by or striking an object, and traffic accidents [3]. Patients suffering from TBI can be divided into three categories according to the Glasgow Coma Scale (GCS). A GCS score of 3~8 is categorized as severe TBI; 9~12 is moderate and 13~15 is mild TBI (mTBI) [4]. Approximately 80~90% of TBI patients are diagnosed with mTBI, and most recover quickly without any treatment. However, a significant minority of patients experience persistent symptoms, which can affect the quality of life [5]. The most common symptoms accompanying mTBI include dizziness, depression, anxiety, and sleep disturbances [6, 7]. These symptoms negatively impact long-term outcomes of mTBI and subsequently increase social and economic burdens [8–11]. Therefore, the identification of crucial factors that influence risk and prognosis of mTBI is urgently needed.

Genetic variations are among the most important determinants of the pathophysiology of brain injury. Dretsch et al. reported that soldiers carrying the brain-derived neurotrophic factor (*BDNF*) Met/Met genotype had a higher incidence of mTBI compared to non-Met/Met carriers [12]. In a prospective cohort study, interleukin-6 receptor (*IL-6R*) and apolipoprotein E (*APOE*) variants were significantly associated with the risk of concussion among 1056 college athletes [13]. A relationship between *TAU* variants and concussion history was also reported in rugby union players [14]. In addition, the effects of genetic variation on the prognosis and clinical outcomes of mTBI were widely described [15, 16].

Insulin-like growth factor 1 (IGF-1) is an important pleiotropic hormone that is involved in various physiological functions. IGF-1 exhibits both neuroprotective and neuroreparative effects following brain injury [17, 18]. Decreased serum IGF-1 levels were reported in TBI patients [19, 20], and in animal studies, circulating IGF-1 was correlated with brain injury-induced cognitive dysfunction and anxiety behaviors [21, 22]. Furthermore, changes in *IGF-1* expression in local brain regions were reported during the acute phase following brain injury [23–25]. Thus, accumulating evidence suggests that IGF-1 plays important roles in the pathophysiology and recovery of brain injury. However, the roles of *IGF-1* variants in mTBI patients have not yet been investigated.

This clinical study attempted to elucidate the effects of *IGF-1* variants on the susceptibility and neuropsychiatric symptoms of mTBI. Based on 176 recruited mTBI patients and 1517 controls from the Taiwan Biobank (TWB) project [26], we compared the genotypic distributions of selected *IGF-1* single nucleotide polymorphisms (SNPs) in mTBI patients and controls. Then, we analyzed associations of these SNPs with the four most common neuropsychiatric symptoms accompanying mTBI: anxiety, depression, dizziness, and sleep disturbances. Further analyses were conducted to explore the SNPs-sex interaction effects on these neuropsychiatric symptoms. In addition, we evaluated the expression levels of *IGF-1* across brain regions, *cis*-expression quantitative trait loci (*cis*-eQTL) and potential functions of the identified SNPs using bioinformatics databases.

## Methods

### Participant recruitment

Patients diagnosed with mTBI in an emergency department (ED) were recruited from three Taipei Medical University (TMU)-affiliated hospitals, including TMU Hospital, Wan Fang Hospital, and Shuang Ho Hospital. The inclusion criteria were that mTBI patients be aged at least 20 years, had an accelerated or decelerated closed injury to the head, and presented to the ED within 6 h of onset of symptoms. The exclusion criteria were as follows: (a) a history of significant ear surgery; (b) a penetrating head injury; (c) pregnancy; (d) a history of dementia or mental disorder; (e) a uremia, liver cirrhosis, heart failure, pulmonary edema, coagulopathy or renal dysfunction; (f) ischemic or hemorrhagic stroke; (g) with an in vivo magnetic implant or pacemaker; and (h) the patient had either died or had already received cardiopulmonary resuscitation before arrival at the ED. Patients with a brain injury caused by abuse or assault were also excluded from the analysis. In addition, the 1517 community-based subjects from the Taiwan Biobank (TWB) were used as our controls. The TWB aims to build a nationwide research database by creating large-scale community-based and hospital-based cohorts in the Taiwanese population [26].

### Study procedures

The mTBI patients were assessed by an emergency medicine specialist. Blood samples and self-reported questionnaires were collected from each patient by a well-trained study nurse in the first week after the mTBI. For the TWB controls, summarized genotype frequency data for each SNP using next-generation sequencing are accessible through Taiwan View (https://taiwanview.twbiobank.org.tw/index). This protocol was approved by the TMU-Joint Institutional Review Board (TMU-JIRB) (nos.: P980803 and 201003008), and written informed consent was received from each mTBI patient.

### Self-reported questionnaires

#### Beck anxiety inventory (BAI)

The BAI is a 21-item self-reported questionnaire that measures the severity of anxiety symptoms. Each item is rated on a 4-point scale from 0 (not at all) to 3 (severely). The total score ranges 0~63, with 0~7 indicating none or minimal, 8~15 mild, 16~25 moderate, and 26~63 severe anxiety. A cutoff score of the BAI of > 7 was used to indicate anxiety symptoms [27].

#### Beck depression inventory (BDI)

The BDI is a 21-item self-reported questionnaire that evaluates the cognitive, behavioral, and physiological symptoms associated with depression. Each item is rated on a 4-point scale from 0 (not depressed) to 3 (severely depressed). The total score ranges 0~63, with 0~9 indicating normal, 10~18 mild, 19~29 moderate, and 30~63 severe depression. A cutoff score of the BDI of > 9 was used to indicate the presence of depressive symptoms [28].

#### Dizziness handicap inventory (DHI)

The DHI is a 25-item self-reported questionnaire to assess the impacts of dizziness on a subject’s quality of life. The DHI has three sub-domains representing functional, emotional, and physical aspects. Each item has three response levels that contribute to the total score which ranges 0~100. A total score of 0~30 indicates a mild, 31~60 a moderate, and 61~100 a severe handicap. A cutoff score of the DHI of > 30 was used to define dizziness-related impairment [29].

#### Pittsburgh sleep quality index (PSQI)

The PSQI is a 19-item self-reported questionnaire that consists of seven domains: sleep quality, sleep latency, sleep duration, sleep efficiency, sleep disturbances, use of sleep medication, and daytime dysfunction. Each domain is scored from 0 (no difficulty) to 3 (severe difficulty). The total score ranges 0~21, with a higher score indicating worse sleep quality. A cutoff score of the PSQI of > 8 was chosen to indicate sleep problems [30, 31].

### Assay of IGF-1

Blood samples were collected from mTBI patients. A radioimmunoassay (RIA) with an IGF-1-RIA-CT (KIP1588) Kit (DIAsource, ImmunoAssays SA, Nivelles, Belgium) was used to verify serum IGF-1 levels of patients in the first week following their brain injury [32].

### DNA extraction

DNA was extracted from peripheral blood samples of recruited mTBI patients. Blood cells were first treated with 0.5% sodium dodecyl sulfate (SDS) lysis buffer, and then a proteinase K solution (1 mg/mL) was applied for 4 h at 60 °C to digest nuclear proteins. Total DNA was harvested using a Gentra extraction kit (Qiagen, Valencia, CA, USA) followed by 70% alcohol precipitation.

### Genotyping of IGF-1

**Fig. 1:**
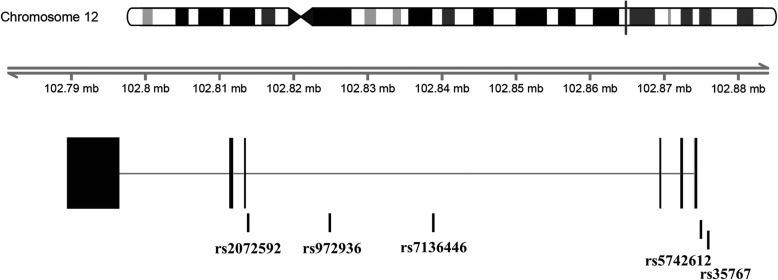
Graphical overview of the genotyped *IGF-1* gene

Five tagging SNPs of *IGF-1* with a minimum allele frequency (MAF) of > 10% were selected from the HapMap Han Chinese database (http://www.hapmap.org/) (Fig. 1). Characteristics of these SNPs are shown in Additional file 1: Table S1. The *IGF-1* SNPs were genotyped using a TaqMan Allelic Discrimination Assay (Applied Biosystems, Foster City, CA, USA). A polymerase chain reaction (PCR) used a 96-well microplate with an ABI 9700 Thermal Cycler (Applied Biosystems). Thermal cycle conditions of the PCR were as follows: denaturing at 95 °C for 10 min, followed by 40 cycles of denaturing at 95 °C for 15 s, and annealing and extension at 60 °C for 1 min. StepOne software (vers. 2.2.2, Applied Biosystems) was used to detect and analyze the fluorescence intensity.

### Functional annotation data query

We queried the tissue-specific expression quantitative trait loci (eQTL) from the GTEx Portal (http://www.gtexportal.org/home/) to evaluate correlations between the SNPs and gene expression profiles [33]. To further assess possible functions of SNPs, bioinformatics databases, including dbSNP, HaploReg V4.1, and RegulomeDB were used to search for the identified SNPs [34–36].

### Claims data from longitudinal health insurance database 2005 (LHID2005)

The data were retrieved from LHID2005 to evaluate the consequent risk of neuropsychiatric symptoms after mTBI in the Taiwanese population. The LHID2005 is a subset that contains one million beneficiaries of the National Health Insurance (NHI) program randomly sampled from the NHI Research Database (NHIRD) in 2005. The mTBI cohort included adults who had a newly diagnosed mTBI identified by ICD-9-CM codes: “skull fracture” (800.0, 800.5, 801.0, 801.5, 803.0, 803.5, 804.0, 804.5), “concussion” (850.0, 850.1, 850.5, 850.9), “intracranial injury of unspecified nature” (854.0), and “head injury, unspecified” (959.01) during 2010. An age- and sex-matched non-mTBI cohort was randomly selected from the remaining participants of the LHID2005. The date of initial diagnosis with mTBI was assigned as the index date. A new diagnosis of anxiety (ICD-9-CM 300), depression (ICD-9-CM 296.*, 300.4, 311), dizziness (ICD-9-CM 780.4), and sleep disorders (ICD-9-CM 780.51, 780.53, 780.57, 307.4, 327.23) during the year following index date were recorded. This protocol was approved by the TMU-JIRB (no.: 201309033).

### RNA sequencing (RNA-seq) data from aging, dementia and TBI study

The available datasets from Aging, Dementia and TBI Study were used to assess the correlation between brain *IGF-1* expression levels and Alzheimer’s disease (AD) among individuals with mild-moderate TBI exposure. The Aging, Dementia and TBI study includes 377 autopsy samples collected from the temporal neocortex (TCx), parietal neocortex (PCx), parietal white matter (FWM), and hippocampus (HIP) of 107 aged brain donors from the Adult Changes in Thought (ACT) study [37]. The RNA integrity number (RIN) corrected, TbT-normalized RNA-seq data was downloaded through the Gene Expression Omnibus (GEO) database (GSE104687). The de-identified clinical metadata was obtained from the Aging, Dementia and TBI Study website (http://aging.brain-map.org/).

### Statistical analysis

R 3.2.0 (http://www.r-project.org/; http://cran.r-project.org/) was used for all statistical analyses. Continuous normally and non-normally distributed variables were respectively presented as the mean ± standard deviation (SD) and median [interquartile range]. Categorical variables were presented as the number (%). The genotypic distributions were assessed for Hardy-Weinberg equilibrium (HWE) using a χ2 goodness-of-fit test. Cochran-Armitage test was used to evaluate differences in genotypic distributions between mTBI patients and controls. A logistic regression under the additive model was performed to estimate associations of SNPs with each phenotype. Age and sex were included as covariates in the regression model. Multiple testing correction was carried out using the Bonferroni correction, and *p*-values of < 0.05 were considered statistically significant. The SNP-sex interaction analysis was performed by adding SNP-by-sex interaction term in the regression model. A chi-square test and two-sample t-test were used respectively to assess the data from LHID2005 and Aging, Dementia and TBI Study.

## Results

### Participant characteristics

In a nationwide population-based analysis, mTBI cohort showed a higher proportion of developing neuropsychiatric symptoms than non-mTBI cohort during the one-year follow-up period (Additional file 1: Figure S1). To elucidate whether the *IGF-1* variation is a potential contributing factor, 176 mTBI patients were recruited. Demographic characteristics were summarized in Table 1. The mean age was 39 (range 20~83) years. Females accounted for 68.8% of the total recruited patients. The main causes of injury were transportation accidents (54.5%) and falls (30.1%). Meanwhile, 1517 subjects from a community-based cohort of the TWB were used as controls in our study. The mean age of controls was 49.5 (range 30~70) years. Females accounted for 49.7%. Most control subjects were recruited from northern and southern Taiwan (Northern: 38.2%, Central: 18.0%, Southern: 42.5%, and Eastern: 1.3%). In the control group, the genotype frequency of each SNP was obtained for analysis.

**Table 1:** Basal characteristics of patients with mild traumatic brain injury (mTBI)

Characteristics	Patients with mTBI
Number of subjects	176
Gender: Female, no. (%)	121 (68.8)
Age (years)^a^	38.80 ± 14.32
Range	20~83
Cause of injury, no. (%)	
Transportation accidents	96 (54.5)
Falls	53 (30.1)
Other	27 (15.3)
GCS^b^	15 [15~15]
GOSE^b^	7 [6~8]
BAI^b^	6 [2~12]
BDI^b^	7 [2~12]
DHI^b^	23 [6~40]
PSQI^b^	6 [5~9]
Serum IGF-1 (ng/mL)^a^	165.80 ± 77.21

^a^mean ± standard deviation. ^b^median [interquartile range]. *GCS* Glasgow Coma Scale, *GOSE* Extended Glasgow Outcome Scale, *BAI* Beck Anxiety Inventory, *BDI* Beck Depression Inventory, *DHI* Dizziness Handicap Inventory, *PSQI* Pittsburgh Sleep Quality Index, *IGF-1* Insulin-like growth factor 1

### Associations between *IGF-1* variants and mTBI susceptibility

We first compared the genotypic distribution of each SNP between mTBI patients and TWB controls. As shown in Table 2, a significant difference in the distribution of rs7136446 was found between the two groups even after multiple testing correction (*p* = 0.008, Bonferroni = 0.040). The C allele of rs7136446 was over-represented in the case group compared to the community-based control group. In addition, rs972936 showed a borderline level of statistical significance (*p* = 0.053, Bonferroni = 0.265). A higher proportion of the T allele of rs972936 was found in mTBI cases compared to the controls.

**Table 2:** Association of insulin-like growth factor 1 (*IGF-1)* variants with susceptibility to mild traumatic brain injury (mTBI)

SNP	Genotype	mTBI patients (*n* = 176)	TWB controls (*n* = 1517)	Cochran-Armitage test	
				*p-value*	Bonferroni
rs35767	GG	75 (42.9)	601 (41.1)	0.964	1.000
	GA	78 (44.6)	698 (47.8)		
	AA	22 (12.6)	162 (11.1)		
rs5742612	AA	78 (44.8)	752 (49.6)	0.193	0.965
	AG	78 (44.8)	635 (41.9)		
	GG	18 (10.3)	128 (8.4)		
rs7136446	TT	107 (60.8)	1049 (69.2)	0.008**	0.040*
	TC	59 (33.5)	426 (28.1)		
	CC	10 (5.7)	41 (2.7)		
rs972936	CC	44 (25.1)	467 (30.8)	0.053	0.265
	CT	88 (50.3)	754 (49.8)		
	TT	43 (24.6)	294 (19.4)		
rs2072592	CC	88 (50.9)	795 (52.4)	0.818	1.000
	CT	72 (41.6)	601 (39.6)		
	TT	13 (7.5)	120 (7.9)		

*indicates *p* < 0.05, **indicates *p* < 0.01

### Associations between *IGF-1* variants and emotional symptoms following mTBI

BAI and BDI scores were respectively used to evaluate anxiety and depressive symptoms. As shown in Table 3, rs972936 showed an association with the BAI score. Patients carrying the T allele had a higher BAI score than that with the C allele. However, significance was not retained after multiple testing correction (*p* = 0.049, Bonferroni = 0.980). In our analysis, there was no significant association between *IGF-1* variants and BDI scores (Table 4).

**Table 3:** Beck Anxiety Inventory (BAI) scores among mild traumatic brain injury (mTBI) patients stratified by different insulin-like growth factor 1 (*IGF-1*) genotypes

SNP	Genotype	BAI score		OR (95% CI)	Additive model^a^	
		BAI ≤7	BAI > 7		*p-*value	Bonferroni
rs35767	GG	44 (43.6)	28 (40.0)	1.12 (0.71–1.76)	0.626	1.000
	GA	44 (43.6)	33 (47.1)			
	AA	13 (12.9)	9 (12.9)			
rs5742612	AA	47 (46.1)	28 (41.2)	1.22 (0.76–1.97)	0.401	1.000
	AG	45 (44.1)	32 (47.1)			
	GG	10 (9.8)	8 (11.8)			
rs7136446	TT	67 (65.7)	37 (52.9)	1.48 (0.89–2.47)	0.129	1.000
	TC	30 (29.4)	28 (40.0)			
	CC	5 (4.9)	5 (7.1)			
rs972936	CC	30 (29.7)	13 (18.6)	1.56 (1.00–2.43)	0.049*	0.980
	CT	50 (49.5)	35 (50.0)			
	TT	21 (20.8)	22 (31.4)			
rs2072592	CC	52 (52.0)	34 (49.3)	1.21 (0.74–1.98)	0.448	1.000
	CT	42 (42.0)	28 (40.6)			
	TT	6 (6.0)	7 (10.1)			

^a^Adjusted for sex and age. *indicates *p* < 0.05. *OR* Odds ratio, *CI* Confidence interval

**Table 4:** Beck Depression Inventory (BDI) scores among mild traumatic brain injury (mTBI) patients stratified by different insulin-like growth factor 1 (*IGF-1*) genotypes

SNP	Genotype	BDI score		OR (95% CI)	Additive model^a^	
		BDI ≤9	BDI > 9		*p-*value	Bonferroni
rs35767	GG	50 (43.1)	23 (41.1)	1.11 (0.70–1.78)	0.655	1.000
	GA	52 (44.8)	25 (44.6)			
	AA	14 (12.1)	8 (14.3)			
rs5742612	AA	51 (44.0)	25 (45.5)	1.06 (0.65–1.73)	0.812	1.000
	AG	54 (46.6)	23 (41.8)			
	GG	11 (9.5)	7 (12.7)			
rs7136446	TT	73 (62.4)	32 (57.1)	1.13 (0.67–1.90)	0.656	1.000
	TC	37 (31.6)	21 (37.5)			
	CC	7 (6.0)	3 (5.4)			
rs972936	CC	27 (23.3)	17 (30.4)	0.96 (0.61–1.50)	0.855	1.000
	CT	62 (53.4)	23 (41.1)			
	TT	27 (23.3)	16 (28.6)			
rs2072592	CC	54 (46.6)	33 (61.1)	0.84 (0.50–1.42)	0.507	1.000
	CT	56 (48.3)	14 (25.9)			
	TT	6 (5.2)	7 (13.0)			

^a^adjusted for sex and age. *OR* Odds ratio, *CI* Confidence interval

### Associations between *IGF-1* variants and dizziness following mTBI

The DHI score was used to interpret the severity of dizziness symptoms, as shown in Table 5. Patients carrying the C allele of rs7136446 had a higher risk of dizziness symptoms than patients carrying the T allele. The statistical significance remained consistent after multiple testing correction (*p* = 0.0004, Bonferroni = 0.008). In addition, patients carrying the T allele of rs972936 also revealed a higher risk of dizziness than those carrying the C allele (*p* = 0.008, Bonferroni = 0.160).

**Table 5:** Dizziness Handicap Inventory (DHI) scores among mild traumatic brain injury (mTBI) patients stratified by different insulin-like growth factor 1 (*IGF-1*) genotypes

SNP	Genotype	DHI score		OR (95% CI)	Additive model^a^	
		DHI ≤30	DHI > 30		*p-*value	Bonferroni
rs35767	GG	47 (42.3)	26 (43.3)	0.89 (0.55–1.44)	0.634	1.000
	GA	49 (44.1)	29 (48.3)			
	AA	15 (13.5)	5 (8.3)			
rs5742612	AA	51 (45.9)	26 (43.3)	0.91 (0.56–1.48)	0.695	1.000
	AG	46 (41.4)	31 (51.7)			
	GG	14 (12.6)	3 (5.0)			
rs7136446	TT	77 (69.4)	28 (45.9)	2.56 (1.49–4.41)	0.0004***	0.008**
	TC	32 (28.8)	25 (41.0)			
	CC	2 (1.8)	8 (13.1)			
rs972936	CC	33 (29.7)	11 (18.3)	1.85 (1.16–2.96)	0.008**	0.160
	CT	58 (52.3)	27 (45.0)			
	TT	20 (18.0)	22 (36.7)			
rs2072592	CC	55 (50.5)	31 (51.7)	0.91 (0.54–1.53)	0.723	1.000
	CT	45 (41.3)	26 (43.3)			
	TT	9 (8.3)	3 (5.0)			

^a^Adjusted for sex and age. **indicates *p* < 0.01, ***indicates *p* < 0.001. *OR* Odds ratio, *CI* Confidence interval

### Associations between *IGF-1* variants and sleep disturbances following mTBI

PHQI total score was used to evaluate the sleep problems of patients who had experienced an mTBI. In the present analysis, we found no statistically significant association between *IGF-1* variants and the PHQI total score (Table 6).

**Table 6:** Pittsburgh Sleep Quality Index (PSQI) scores among mild traumatic brain injury (mTBI) patients stratified by different insulin-like growth factor 1 (*IGF-1*) genotypes

SNP	Genotype	PSQI score		OR (95% CI)	Additive model^a^	
		PSQI ≤8	PSQI > 8		*p-*value	Bonferroni
rs35767	GG	44 (40.4)	24 (49.0)	0.84 (0.50–1.41)	0.508	1.000
	GA	53 (48.6)	19 (38.8)			
	AA	12 (11.0)	6 (12.2)			
rs5742612	AA	48 (44.0)	23 (47.9)	0.94 (0.55–1.60)	0.820	1.000
	AG	51 (46.8)	20 (41.7)			
	GG	10 (9.2)	5 (10.4)			
rs7136446	TT	70 (64.2)	25 (50.0)	1.52 (0.88–2.60)	0.131	1.000
	TC	33 (30.3)	21 (42.0)			
	CC	6 (5.5)	4 (8.0)			
rs972936	CC	28 (25.7)	11 (22.4)	1.38 (0.85–2.26)	0.191	1.000
	CT	59 (54.1)	22 (44.9)			
	TT	22 (20.2)	16 (32.7)			
rs2072592	CC	53 (49.5)	27 (55.1)	0.94 (0.54–1.65)	0.839	1.000
	CT	48 (44.9)	18 (36.7)			
	TT	6 (5.6)	4 (8.2)			

^a^Adjusted for sex and age. *OR* Odds ratio, *CI* Confidence interval

### The sex-specific effects of *IGF-1* variants following mTBI

One hundred seventy-six mTBI patients were further stratified to evaluate the sex-specific effects of *IGF-1* variants on neuropsychiatric symptoms (Additional file 1: Table S2). Patients carrying the minor alleles of rs972936 and rs7136446 showed a higher DHI score in males and females, respectively (rs972936 in males: *p* = 0.037, Bonferroni = 0.740; rs7136446 in females: *p* = 0.001, Bonferroni = 0.020) (Additional file 1: Table S5). Meanwhile, patients carrying the minor allele of rs7136446 showed a higher PSQI score in females, but not in males (rs7136446 in females: *p* = 0.035, Bonferroni = 0.700) (Additional file 1: Table S6). To confirm these findings, the interaction analyses between SNPs and sex were performed to elucidate whether the impacts of rs972936 and rs7136446 were significantly different between males and females. However, we found no significant interaction effects of these SNPs with sex on the neuropsychiatric symptoms following mTBI (Additional file 1: Table S7).

### Associations between *IGF-1* variants and multiple neuropsychiatric symptoms

Having multiple neuropsychiatric symptoms would make recovery more difficult for mTBI patients. Therefore, we compared the genotypic distributions between patients with all four neuropsychiatric symptoms (*n* = 18) and patients without any symptoms (*n* = 61). The characteristics are shown in Table 7. The genotypic distributions of both rs7136446 and rs972936 showed a difference between the two groups. The C allele of rs7136446 and the T allele of rs972936 were over-represented in the group with multiple neuropsychiatric symptoms (rs7136446: *p* = 0.005, Bonferroni = 0.025; rs972936: *p* = 0.041, Bonferroni = 0.205) (Table 8).

**Table 7:** Basal characteristics of mild traumatic brain injury (mTBI) patients stratified by the presence or absence of multiple neuropsychiatric symptoms

Characteristics	Patients without any neuropsychiatric symptoms^c^	Patients with multiple neuropsychiatric symptoms^c^
Number of subjects	61	18
Gender: Female, no. (%)	39(63.9)	16 (88.9)
Age (years)^a^	37.21 ± 15.04	42.22 ± 16.06
Range	20~83	20~75
Cause of injury, no. (%)		
Transportation accidents	31 (50.8)	8 (44.4)
Falls	22 (36.1)	7 (38.9)
Other	8 (13.1)	3 (16.7)
GCS^b^	15 [15~15]	15 [15~15]
GOSE^b^	8 [7~8]	6 [6~7]
BAI^b^	2 [1~4]	18 [12~26.75]
BDI^b^	2 [1~5]	17.5 [14.25~19]
DHI^b^	4 [0~16]	52 [38.5~65.5]
PSQI^b^	5 [4~6]	11 [10~14]
Serum IGF-1 (ng/mL)^a^	177.7 ± 83.40	147.4 ± 69.87

^a^mean ± standard deviation. ^b^median [interquartile range]. *GCS* Glasgow Coma Scale, *GOSE* Extended Glasgow Outcome Scale, *BAI* Beck Anxiety Inventory, *BDI* Beck Depression Inventory, *DHI* Dizziness Handicap Inventory, *PSQI* Pittsburgh Sleep Quality Index, *IGF-1* Insulin-like growth factor 1. ^c^Patients without any neuropsychiatric symptoms were indicated by lower scores on all questionnaires (BAI ≤7, BDI ≤9, DHI ≤30, and PSQI ≤8); Patients with multiple neuropsychiatric symptoms were indicated by higher scores on all questionnaires (BAI > 7, BDI > 9, DHI > 30, and PSQI > 8)

**Table 8:** Multiple neuropsychiatric symptoms of mild traumatic brain injury (mTBI) patients stratified by different insulin-like growth factor-1 (*IGF-1*) genotypes

SNP	Genotype	Patients without any neuropsychiatric symptoms (*n* = 61)	Patients with multiple neuropsychiatric symptoms (*n* = 18)	OR (95% CI)	Additive model^a^	
					*p*-value	Bonferroni
rs35767	GG	27 (44.3)	9 (50.0)	0.79 (0.36–1.75)	0.557	1.000
	GA	26 (42.6)	7 (38.9)			
	AA	8 (13.1)	2 (11.1)			
rs5742612	AA	29 (47.5)	9 (52.9)	0.71 (0.31–1.65)	0.420	1.000
	AG	24 (39.3)	7 (41.2)			
	GG	8 (13.1)	1 (5.9)			
rs7136446	TT	40 (65.6)	5 (27.8)	3.73 (1.42–9.82)	0.005**	0.025*
	TC	19 (31.1)	11 (61.1)			
	CC	2 (3.3)	2 (11.1)			
rs972936	CC	15 (24.6)	1 (5.6)	2.58 (1.00–6.67)	0.041*	0.205
	CT	37 (60.7)	11 (61.1)			
	TT	9 (14.8)	6 (33.3)			
rs2072592	CC	29 (48.3)	10 (58.8)	0.72 (0.27–1.89)	0.493	1.000
	CT	28 (46.7)	6 (35.3)			
	TT	3 (5.0)	1 (5.9)			

^a^Adjusted for sex and age. *indicates *p* < 0.05, **indicates *p* < 0.01. *OR* Odds ratio, *CI* Confidence interval

### eQTLs and functional annotation

The eQTLs for the two most significant SNPs were accessed from the GTEx Portal. After the multiple testing correction, rs731446 showed an ability to alter the expression of WASH complex subunit 3 (*WASHC3*) in several tissues. rs972936 can alter the expression of poly (ADP-ribose) polymerase 1 (PARP-1) - binding protein (*PARPBP*) and *WASHC3* across different types of tissues (Additional file 1: Table S8). Results of functional annotations using HaploReg V4.1 and Regulome DB are shown in Additional file 1: Table S9.

### Association between *IGF-1* expression and Alzheimer’s disease (AD) after mTBI

We further search the corrections of rs972936 and rs7136446 with *IGF-1* expressions across 13 brain tissue types through GTEx with a *p*-value less than 0.05. As shown in Additional file 1: Table S10, the risk alleles of both SNPs in the present study showed lower levels of *IGF-1* expression in the hippocampus, an important brain region for learning and memory. We therefore evaluate the effects of hippocampal *IGF-1* expression on AD among individuals with TBI exposure. The RNA-seq data of 107 older brain donors from Aging, Dementia, and TBI Study were retrieved. In total, 53 donors had a history of mild-moderate TBI with loss of consciousness. Of the 53 donors, 14 died with a clinical diagnosis of AD and 26 died with no dementia (Table 9). The levels of *IGF-1* expression were significantly different between the two groups in the hippocampus (*p* = 0.008) and parietal cortex (*p* = 0.019) (Table 10). In the donors without TBI exposure, we find no statistically significant differences of *IGF-1* expression levels between two groups (Additional file 1: Table S11).

**Table 9:** Characteristics of brain donors with a history of traumatic brain injury (TBI) exposure stratified by the presence or absence of Alzheimer’s disease (AD) from GEO database (GSE104687)

	Alzheimer’s disease^c^ (*n* = 14)	No Dementia^c^ (*n* = 26)
Number of subjects	14	26
Gender: Female, no. (%)	8 (57.1)	7 (26.9)
Education (years) ^a^	14.14 ± 3.74	14.58 ± 3.41
Age at death, no. (%)		
> 100	3 (21.4)	1 (3.8)
95–99	0 (0)	7 (26.9)
90–94	3 (21.4)	5 (19.2)
85–89	4 (28.6)	7 (26.9)
80–84	3 (21.4)	2 (7.7)
75–79	1 (7.1)	4 (15.4)
Number of TBIs, no. (%)		
1	11 (78.6)	20 (76.9)
2	2 (14.3)	5 (19.2)
3	1 (7.1)	1 (3.8)
CERAD score ^b^	2 [1~2.75]	1 [1~2]
BRAAK stage ^b^	5 [3.25~6]	3 [1.25~4]
NIA Reagan ^b^	2 [1~2.75]	1 [1~2]
APOE ε4 alleles, no. (%)		
Yes	3 (21.4)	4 (15.4)
No	10 (71.4)	21 (80.8)
Unknown	1 (7.1)	1 (3.8)

^a^mean ± standard deviation. ^b^median [interquartile range]. *CERAD* Consortium to Establish a Registry for Alzheimer’s Disease, *NIA* National Institute on Aging, *APOE* Apolipoprotein E. ^c^The diagnosis was based on the DSM IV clinical diagnosis

**Table 10:** Association between insulin-like growth factor 1 (*IGF-1*) expression levels and Alzheimer’s disease (AD) in brain donors with a history of traumatic brain injury (TBI)

Brain region	Alzheimer’s disease			No Dementia			*p*-value
	Samples	Mean	SD	Samples	Mean	SD	
Parietal white matter (FWM)	13	0.702	0.172	20	0.722	0.227	0.776
Parietal cortex (PCx)	13	0.969	0.143	19	1.120	0.203	0.019*
Temporal cortex (TCx)	14	1.044	0.248	21	1.102	0.204	0.480
Hippocampus (HIP)	12	1.196	0.206	21	1.475	0.359	0.008**

*SD* Standard deviation. *indicates *p* < 0.05, **indicates *p* < 0.01

## Discussion

The IGF-1 signaling pathway is involved in numerous brain diseases [38]. IGF-1 binding to its receptor (IGF-1R) initiates intracellular signaling that subsequently activates two major pathways: phosphatidyl-inositol 3-kinase/ protein kinase B (PI3K/AKT) and mitogen-activated protein kinase/ extracellular signal-regulated kinase (MAPK/ERK). Activation of these pathways modulates various brain functions, such as glucose utilization, neurogenesis, synaptic plasticity, and angiogenesis [18]. These functions may then influence primary responses of an individual’s brain after being struck by an external force as well as the secondary damage following a brain injury.

In this study, we found that *IGF-1* variants (rs7136446 and rs972936) were associated with the susceptibility and neuropsychiatric symptoms of mTBI. The minor allele of rs7136446 was over-represented in mTBI patients compared to a community-based control population. In addition, patients carrying the minor alleles of these two SNPs showed more dizziness and multiple neuropsychiatric symptoms in the first week after brain injury. These early neuropsychiatric symptoms largely reflect a disturbance of brain functions and could also predict long-term consequences following mTBI [10, 39–41]. Our findings support the idea that *IGF-1* variants are important factors for the pathophysiology of mTBI.

In addition, the minor alleles of rs7136446 and rs972936 showed lower *IGF-1* expressions in the hippocampus. Meanwhile, brain donors diagnosed with AD also showed lower levels of hippocampal *IGF-1* expression than those with no dementia among individuals with TBI exposure. These findings provide additional clues that *IGF-1* variation may not only affects early neuropsychiatric symptoms but also neurodegeneration after brain injury. The regulatory effects of IGF-1 signaling on amyloid-β (Aβ) deposition and tau phosphorylation were reported in previous animal studies [42, 43]. In addition, hypoxia repressed the activities of IGF-1 signaling in zebrafish embryos [44, 45]. IGF-1 signaling in human astrocytes displayed the capacity to protect neurons from oxidative stress [46]. These pathophysiological processes had also been reported as the possible link between TBI and AD [47].

In the subgroup analyses by gender, the results indicated that rs972936 and rs7136446 may exert more influence on males and females, respectively. However, in the SNPs-sex interaction analyses, we find no significant interaction between each SNP and gender. It seems that gender may not the strong co-regulator participated in the correlations between *IGF-1* variants and early neuropsychiatric symptoms after mTBI. However, previous studies revealed that the effects of *IGF-1* variants on the risk of some cancers were modified by sex or menopausal status [48, 49]. In addition, the cross-interactions between estrogen and IGF-1 signaling were widely reported in the brain [50, 51]. The estrogen and IGF-1 co-regulated the PI3K/AKT and MAPK/ERK pathways which stimulate the adult neurogenesis and promote the neuroprotection in the brain regions [52]. Therefore, even though we didn’t find the strong gender-specific effects of SNPs chosen for the present study, the associations between sex hormones, *IGF-1*, and mTBI are worth further investigation.

SNPs rs7136446 and rs972936 have been studied in several human traits and diseases. Huuskonen et al. found that rs7136446 was associated with maximal force production and body composition [53], while Aberg et al. demonstrated that patients carrying the major allele of rs7136446 showed favorable functional outcomes after ischemic stroke [54]. In addition, the rs972936 SNP was significantly associated with susceptibility to Alzheimer’s disease and Parkinson’s disease in a Han Chinese population [55, 56]. Although both rs7136446 and rs972936 are located in intronic regions of *IGF-1*, these reports provide evidence that the alleles indeed influence human traits and diseases. Functional annotations using bioinformatics databases revealed that rs7136446 and rs972936 can alter the expression level of *WASHC3*, also known as Coiled-coil domain-containing protein 53 (*CCDC53*). Interestingly, a previous animal study showed that *Ccdc53* expression exhibited a maximum negative fold-change (− 2.42) in mTBI induced hippocampal gene expression profiles compared to a sham-operated group [57, 58]. Moreover, these SNPs are located in a region with known histone modifications, DNAse, and regulatory motifs. Furthermore, evidence from recent studies supports the involvement of epigenetic modulations following brain injury which can further affect the recovery after mTBI [59, 60]. Taken together, these studies provide tantalizing clues as to the possible mechanisms that could underlie the connection between the identified SNPs and mTBI pathophysiology.

There are several limitations to our study. First, we used 1517 community-based control subjects from the TWB project as our comparison group. Summary statistics of the SNPs were accessed through the Taiwan View website. However, previous injury histories of these subjects were lacking, so we cannot exclude the possibility that some subjects may have experienced an mTBI in the past. Nevertheless, the present findings still offer important information about the effects of *IGF-1* on the risk of mTBI. Well-designed prospective studies that include large sample sizes of both mTBI cases and controls are needed to fully elucidate corrections between *IGF-1* variants and mTBI. Second, we used a candidate gene approach to evaluate the roles of *IGF-1* variants in mTBI outcomes. However, the influence of variants in other unexamined genes cannot be ruled out. Since the pathophysiology of mTBI depends on complex regulation by multiple signaling pathways, a genome-wide approach may yield further insights into genetic variations and prognoses of mTBI.

## Conclusions

In this study, we found associations of *IGF-1* variants with susceptibility and neuropsychiatric outcomes of mTBI, highlighting the important roles of *IGF-1* in the pathophysiology of mTBI. Further studies focusing on the IGF-1 signaling pathway are needed to elucidate the mechanism underlying this association. The role of epigenetic modulations in the risk of mTBI is especially important for further investigation.

## Supplementary information

**Additional file 1: Figure S1.** Proportion of subjects developing neuropsychiatric symptoms among mTBI and non-mTBI cohorts during the 1-year follow-up period from the index date. **Table S1.** The allele frequency of single nucleotide polymorphisms (SNPs) in different ethnic groups. **Table S2.** Basal characteristics of patients with mild traumatic brain injury (mTBI) stratified by sex. **Table S3.** Sex-stratified analyses for Beck Anxiety Inventory (BAI) score. **Table S4.** Sex-stratified analyses for Beck Depression Inventory (BDI) score. **Table S5.** Sex-stratified analyses for Dizziness Handicap Inventory (DHI) score. **Table S6.** Sex-stratified analyses for Pittsburgh Sleep Quality Index (PSQI) score. **Table S7.** Single nucleotide polymorphisms (SNPs)-sex interaction analyses for neuropsychiatric symptoms following mild traumatic brain injury (mTBI). **Table S8.** Expression quantitative trail loci (eQTL) results from Genotype-tissue expression (GTEx). **Table S9.** Functional annotation by HaploReg V4.1 and RegulomeDB. **Table S10.** Expression quantitative trait loci (eQTL) results of rs7136446 and rs972936 with insulin-like growth factor 1 *(IGF-1*) expressions in brain tissues from Genotype-tissue expression (GTEx). **Table S11.** Association between insulin-like growth factor 1 (*IGF-1*) expression levels and Alzheimer’s disease (AD) in brain donors with no history of traumatic brain injury (TBI).

## Data Availability

The datasets used and/or analyzed during the current study are available from the corresponding author on reasonable request.

## Abbreviations

AD: Alzheimer’s disease
BAI: Beck Anxiety Inventory
BDI: Beck Depression Inventory
CCDC53: Coiled-coil domain-containing 53
DHI: Dizziness Handicap Inventory
ED: Emergency department
eQTL: Expression quantitative trait loci
GCS: Glasgow Coma Scale
GTEx: Genotype-tissue expression
IGF-1: Insulin-like growth factor 1
LHID2005: Longitudinal Health Insurance Database 2005
MAPK/ERK: Mitogen-activated protein kinase/ extracellular signal-regulated kinase
mTBI: Mild traumatic brain injury
PI3K/AKT: Phosphatidyl-inositol 3-kinase/ protein kinase B
PSQI: Pittsburgh Sleep Quality Index
SNP: Single-nucleotide polymorphism
TBI: Traumatic brain injury
TWB: Taiwan Biobank
WASHC3: WASH complex subunit 3

## References

[1] James SL, Theadom A, Ellenbogen RG, Bannick MS, Montjoy-Venning W, Lucchesi LR, et al. Global, regional, and national burden of traumatic brain injury and spinal cord injury, 1990–2016: a systematic analysis for the Global Burden of Disease Study 2016. Lancet Neurol. 2019;18(1):56–87. 10.1016/S1474-4422(18)30415-0 PMC6291456 30497965

[2] McKee AC, Daneshvar DH (2015). The neuropathology of traumatic brain injury. Handb Clin Neurol.

[3] Asemota AO, George BP, Bowman SM, Haider AH, Schneider EB (2013). Causes and trends in traumatic brain injury for United States adolescents. J Neurotrauma.

[4] Teasdale G, Jennett B (1974). Assessment of coma and impaired consciousness. A practical scale. Lancet (London, England).

[5] Blennow K, Brody DL, Kochanek PM, Levin H, McKee A, Ribbers GM (2016). Traumatic brain injuries. Nat Rev Dis Prim.

[6] Prince Carolyn, Bruhns Maya (2017). Evaluation and Treatment of Mild Traumatic Brain Injury: The Role of Neuropsychology. Brain Sciences.

[7] Eme Robert (2017). Neurobehavioral Outcomes of Mild Traumatic Brain Injury: A Mini Review. Brain Sciences.

[8] Iverson GL, Gardner AJ, Terry DP, Ponsford JL, Sills AK, Broshek DK (2017). Predictors of clinical recovery from concussion: a systematic review. Br J Sports Med.

[9] Chan LG, Feinstein A (2015). Persistent sleep disturbances independently predict poorer functional and social outcomes 1 year after mild traumatic brain injury. J Head Trauma Rehabil.

[10] Chamelian L, Feinstein A (2004). Outcome after mild to moderate traumatic brain injury: the role of dizziness. Arch Phys Med Rehabil.

[11] van der Naalt J, Timmerman ME, de Koning ME, van der Horn HJ, Scheenen ME, Jacobs B (2017). Early predictors of outcome after mild traumatic brain injury (UPFRONT): an observational cohort study. Lancet Neurol.

[12] Dretsch MN, Williams K, Emmerich T, Crynen G, Ait-Ghezala G, Chaytow H (2016). Brain-derived neurotropic factor polymorphisms, traumatic stress, mild traumatic brain injury, and combat exposure contribute to postdeployment traumatic stress. Brain Behav.

[13] Terrell TR, Abramson R, Barth JT, Bennett E, Cantu RC, Sloane R (2018). Genetic polymorphisms associated with the risk of concussion in 1056 college athletes: a multicentre prospective cohort study. Br J Sports Med.

[14] Abrahams S, Mc Fie S, Patricios J, Suter J, September AV, Posthumus M (2019). Toxic TAU: the TAU gene polymorphisms associate with concussion history in rugby union players. J Sci Med Sport.

[15] Bennett ER, Reuter-Rice K, Laskowitz DT, Laskowitz D, Grant G (2016). Frontiers in neuroscience genetic influences in traumatic brain injury. Translational research in traumatic brain injury.

[16] Davidson J, Cusimano MD, Bendena WG (2015). Post-traumatic brain injury: genetic susceptibility to outcome. Neuroscientist.

[17] Madathil SK, Saatman KE, Kobeissy FH (2015). Frontiers in neuroengineering IGF-1/IGF-R signaling in traumatic brain injury: impact on cell survival, neurogenesis, and behavioral outcome. Brain neurotrauma: molecular, neuropsychological, and rehabilitation aspects.

[18] Mangiola A, Vigo V, Anile C, De Bonis P, Marziali G, Lofrese G (2015). Role and importance of IGF-1 in traumatic brain injuries. Biomed Res Int.

[19] Sanus GZ, Tanriverdi T, Coskun A, Hanimoglu H, Is M, Uzan M (2007). Cerebrospinal fluid and serum levels of insulin-like growth factor-1 and insulin-like growth factor binding protein-3 in patients with severe head injury. Ulus Travma Acil Cerrahi Dergisi = Turkish J Trauma Emerg Surg.

[20] Wagner J, Dusick JR, McArthur DL, Cohan P, Wang C, Swerdloff R (2010). Acute gonadotroph and somatotroph hormonal suppression after traumatic brain injury. J Neurotrauma.

[21] Ozdemir D, Baykara B, Aksu I, Kiray M, Sisman AR, Cetin F (2012). Relationship between circulating IGF-1 levels and traumatic brain injury-induced hippocampal damage and cognitive dysfunction in immature rats. Neurosci Lett.

[22] Baykara B, Aksu I, Buyuk E, Kiray M, Sisman AR, Baykara B (2013). Progesterone treatment decreases traumatic brain injury induced anxiety and is correlated with increased serum IGF-1 levels; prefrontal cortex, amygdala, hippocampus neuron density; and reduced serum corticosterone levels in immature rats. Biotech Histochem.

[23] Madathil SK, Evans HN, Saatman KE (2010). Temporal and regional changes in IGF-1/IGF-1R signaling in the mouse brain after traumatic brain injury. J Neurotrauma.

[24] Schober ME, Block B, Beachy JC, Statler KD, Giza CC, Lane RH (2010). Early and sustained increase in the expression of hippocampal IGF-1, but not EPO, in a developmental rodent model of traumatic brain injury. J Neurotrauma.

[25] Walter HJ, Berry M, Hill DJ, Logan A (1997). Spatial and temporal changes in the insulin-like growth factor (IGF) axis indicate autocrine/paracrine actions of IGF-I within wounds of the rat brain. Endocrinology.

[26] Chen CH, Yang JH, Chiang CWK, Hsiung CN, Wu PE, Chang LC (2016). Population structure of Han Chinese in the modern Taiwanese population based on 10,000 participants in the Taiwan biobank project. Hum Mol Genet.

[27] Beck AT, Epstein N, Brown G, Steer RA (1988). An inventory for measuring clinical anxiety: psychometric properties. J Consult Clin Psychol.

[28] Beck AT, Ward CH, Mendelson M, Mock J, Erbaugh J (1961). An inventory for measuring depression. Arch Gen Psychiatry.

[29] Jacobson GP, Newman CW (1990). The development of the dizziness handicap inventory. Arch Otolaryngol Head Neck Surg.

[30] Buysse DJ, Reynolds CF, Monk TH, Berman SR, Kupfer DJ (1989). The Pittsburgh sleep quality index: a new instrument for psychiatric practice and research. Psychiatry Res.

[31] Fictenberg NL, Putnam SH, Mann NR, Zafonte RD, Millard AE (2001). Insomnia screening in postacute traumatic brain injury: utility and validity of the Pittsburgh sleep quality index. Am J Phys Med Rehabil.

[32] Sung CW, Chen KY, Chiang YH, Chiu WT, Ou JC, Lee HC (2016). Heart rate variability and serum level of insulin-like growth factor-1 are correlated with symptoms of emotional disorders in patients suffering a mild traumatic brain injury. Clin Neurophysiol.

[33] Lonsdale J, Thomas J, Salvatore M, Phillips R, Lo E, Shad S, et al. The Genotype-Tissue Expression (GTEx) project. Nat Genet. 2013;45(6):580–5. 10.1038/ng.2653 PMC4010069 23715323

[34] Ward LD, Kellis M (2016). HaploReg v4: systematic mining of putative causal variants, cell types, regulators and target genes for human complex traits and disease. Nucleic Acids Res.

[35] Boyle AP, Hong EL, Hariharan M, Cheng Y, Schaub MA, Kasowski M (2012). Annotation of functional variation in personal genomes using RegulomeDB. Genome Res.

[36] Sherry ST, Ward MH, Kholodov M, Baker J, Phan L, Smigielski EM (2001). dbSNP: the NCBI database of genetic variation. Nucleic Acids Res.

[37] Miller JA, Guillozet-Bongaarts A, Gibbons LE, Postupna N, Renz A, Beller AE, et al. Neuropathological and transcriptomic characteristics of the aged brain. eLife. 2017;6:e31126. 10.7554/eLife.31126 PMC5679757 29120328

[38] Trejo JL, Carro E, Garcia-Galloway E, Torres-Aleman I (2004). Role of insulin-like growth factor I signaling in neurodegenerative diseases. J Mol Med (Berlin, Germany).

[39] Lau BC, Kontos AP, Collins MW, Mucha A, Lovell MR (2011). Which on-field signs/symptoms predict protracted recovery from sport-related concussion among high school football players?. Am J Sports Med.

[40] Dischinger PC, Ryb GE, Kufera JA, Auman KM (2009). Early predictors of postconcussive syndrome in a population of trauma patients with mild traumatic brain injury. J Trauma.

[41] Rao V, McCann U, Han D, Bergey A, Smith MT (2014). Does acute TBI-related sleep disturbance predict subsequent neuropsychiatric disturbances?. Brain Inj.

[42] Cheng CM, Tseng V, Wang J, Wang D, Matyakhina L, Bondy CA (2005). Tau is hyperphosphorylated in the insulin-like growth factor-I null brain. Endocrinology.

[43] Giuffrida ML, Tomasello F, Caraci F, Chiechio S, Nicoletti F, Copani A (2012). Beta-amyloid monomer and insulin/IGF-1 signaling in Alzheimer's disease. Mol Neurobiol.

[44] Kamei H, Ding Y, Kajimura S, Wells M, Chiang P, Duan C (2011). Role of IGF signaling in catch-up growth and accelerated temporal development in zebrafish embryos in response to oxygen availability. Dev (Cambridge, England).

[45] Kajimura S, Aida K, Duan C (2005). Insulin-like growth factor-binding protein-1 (IGFBP-1) mediates hypoxia-induced embryonic growth and developmental retardation. Proc Natl Acad Sci U S A.

[46] Ratcliffe LE, Vazquez Villasenor I, Jennings L, Heath PR, Mortiboys H, Schwartzentruber A (2018). Loss of IGF1R in human astrocytes alters complex I activity and support for neurons. Neuroscience.

[47] Ramos-Cejudo J, Wisniewski T, Marmar C, Zetterberg H, Blennow K, de Leon MJ (2018). Traumatic brain injury and Alzheimer's disease: the cerebrovascular link. EBioMedicine.

[48] Shi J, Aronson KJ, Grundy A, Kobayashi LC, Burstyn I, Schuetz JM (2016). Polymorphisms of insulin-like growth factor 1 pathway genes and breast Cancer risk. Front Oncol.

[49] Ong J, Salomon J, te Morsche RH, Roelofs HM, Witteman BJ, Dura P (2014). Polymorphisms in the insulin-like growth factor axis are associated with gastrointestinal cancer. PLoS One.

[50] Huffman J, Hoffmann C, Taylor GT (2017). Integrating insulin-like growth factor 1 and sex hormones into neuroprotection: implications for diabetes. World J Diabetes.

[51] Garcia-Segura LM, Arevalo MA, Azcoitia I (2010). Interactions of estradiol and insulin-like growth factor-I signalling in the nervous system: new advances. Prog Brain Res.

[52] Sohrabji F (2015). Estrogen-IGF-1 interactions in neuroprotection: ischemic stroke as a case study. Front Neuroendocrinol.

[53] Huuskonen A, Lappalainen J, Oksala N, Santtila M, Hakkinen K, Kyrolainen H (2011). Common genetic variation in the IGF1 associates with maximal force output. Med Sci Sports Exerc.

[54] Aberg ND, Olsson S, Aberg D, Jood K, Stanne TM, Nilsson M (2013). Genetic variation at the IGF1 locus shows association with post-stroke outcome and to circulating IGF1. Eur J Endocrinol.

[55] Wang W, Yu JT, Tan L, Liu QY, Wang HF, Ma XY (2012). Insulin-like growth factor 1 (IGF1) polymorphism is associated with Alzheimer's disease in Han Chinese. Neurosci Lett.

[56] Xiao Yousheng, Cen Luan, Mo Mingshu, Chen Xiang, Huang Shuxuan, Wei Lei, Li Shaomin, Yang Xinling, Qu Shaogang, Pei Zhong, Xu Pingyi (2017). Association ofIGF1gene polymorphism with Parkinson's disease in a Han Chinese population. The Journal of Gene Medicine.

[57] Tweedie D, Rachmany L, Rubovitch V, Zhang Y, Becker KG, Perez E (2013). Changes in mouse cognition and hippocampal gene expression observed in a mild physical- and blast-traumatic brain injury. Neurobiol Dis.

[58] Tweedie D, Rachmany L, Kim DS, Rubovitch V, Lehrmann E, Zhang Y (2016). Mild traumatic brain injury-induced hippocampal gene expressions: the identification of target cellular processes for drug development. J Neurosci Methods.

[59] Wong VS, Langley B (2016). Epigenetic changes following traumatic brain injury and their implications for outcome, recovery and therapy. Neurosci Lett.

[60] Schober ME, Ke X, Xing B, Block BP, Requena DF, McKnight R (2012). Traumatic brain injury increased IGF-1B mRNA and altered IGF-1 exon 5 and promoter region epigenetic characteristics in the rat pup hippocampus. J Neurotrauma.

