# Supplementary material for: The functional roles of IGF-1 variants in the susceptibility and clinical outcomes of mild traumatic brain injury
Source: J Biomed Sci. 2019 Dec 2;26:94. doi: 10.1186/s12929-019-0587-9 (PMC6886173; doi:10.1186/s12929-019-0587-9)
Supplement: Supplementary file 1 — Additional file 1: Figure S1. Proportion of subjects developing neuropsychiatric symptoms among mTBI and non-mTBI cohorts during the 1-year follow-up period from the index date. Table S1. The allele frequency of single nucleotide polymorphisms (SNPs) in different ethnic groups. Table S2. Basal characteristics of patients with mild traumatic brain injury (mTBI) stratified by sex. Table S3. Sex-stratified analyses for Beck Anxiety Inventory (BAI) score. Table S4. Sex-stratified analyses for Beck Depression Inventory (BDI) score. Table S5. Sex-stratified analyses for Dizziness Handicap Inventory (DHI) score. Table S6. Sex-stratified analyses for Pittsburgh Sleep Quality Index (PSQI) score. Table S7. Single nucleotide polymorphisms (SNPs)-sex interaction analyses for neuropsychiatric symptoms following mild traumatic brain injury (mTBI). Table S8. Expression quantitative trail loci (eQTL) results from Genotype-tissue expression (GTEx). Table S9. Functional annotation by HaploReg V4.1 and RegulomeDB. Table S10. Expression quantitative trait loci (eQTL) results of rs7136446 and rs972936 with insulin-like growth factor 1 (IGF-1) expressions in brain tissues from Genotype-tissue expression (GTEx). Table S11. Association between insulin-like growth factor 1 (IGF-1) expression levels and Alzheimer’s disease (AD) in brain donors with no history of traumatic brain injury (TBI). [file 12929_2019_587_MOESM1_ESM.docx]

**Figure S1. Proportion of subjects developing neuropsychiatric symptoms among mTBI and non-mTBI cohorts during the 1-year follow-up period from the index date**


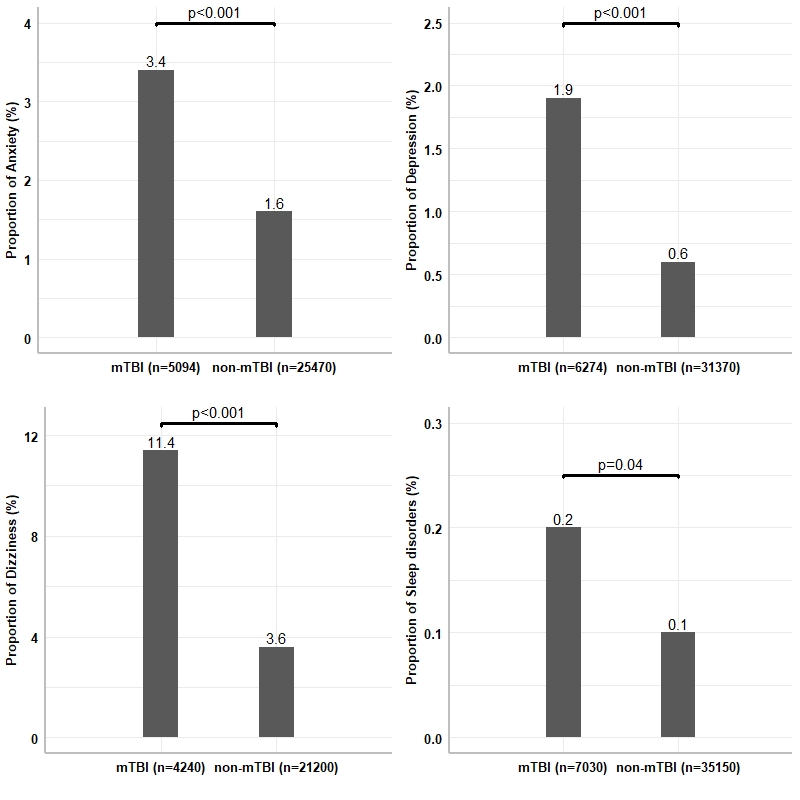


| **Table S1. The allele frequency of single nucleotide polymorphisms (SNPs) in different ethnic groups** | | | | | | | | | | | | | | |  | |
| --- | --- | --- | --- | --- | --- | --- | --- | --- | --- | --- | --- | --- | --- | --- | --- | --- |
| **Gene** | **Position** (**GRCh37)** | **SNP** |  | **Allele** | |  | **AFR**  **freq** | **AMR**  **freq** | **ASN**  **freq** | **EUR**  **freq** | **TWB controls** | |  | **mTBI patients** | | |
|  |  |  |  | **Ref** | **Alt** |  |  |  |  |  | **freq** | **HWE** |  | **freq** | | **HWE** |
| *IGF1* | chr12:102875569 | rs35767 |  | G | A |  | 0.42 | 0.25 | 0.34 | 0.16 | 0.35 | 0.054 |  | 0.35 | | 0.806 |
|  | chr12:102874864 | rs5742612 |  | A | G |  | 0.02 | 0.1 | 0.29 | 0.05 | 0.29 | 0.710 |  | 0.33 | | 0.817 |
|  | chr12:102838515 | rs7136446 |  | T | C |  | 0.3 | 0.23 | 0.19 | 0.39 | 0.17 | 0.774 |  | 0.22 | | 0.623 |
|  | chr12:102824921 | rs972936 |  | C | T |  | 0.4 | 0.26 | 0.44 | 0.25 | 0.44 | 0.740 |  | 0.50 | | 0.939 |
|  | chr12:102813632 | rs2072592 |  | C | T |  | 0 | 0.09 | 0.25 | 0.02 | 0.28 | 0.666 |  | 0.28 | | 0.742 |
| *AFR* African American, *AMR*, Americas, *ASN* Asian, *EUR* European, *TWB* Taiwan Biobank, Freq: frequency shows as Alt allele. mTBI: mild traumatic brain injury patients recruited in our study. HWE: *p*-value for Hardy-Weinberg equilibrium. | | | | | | | | | | | | | | | | |

| **Table S2. Basal characteristics of patients with mild traumatic brain injury (mTBI) stratified by sex** | | |
| --- | --- | --- |
| **Characteristics** | **Males** | **Females** |
| Number of subjects | 55 | 121 |
| Age (years)^a^ | 37.98 ± 15.46 | 39.17 ± 13.83 |
| Range | 20-83 | 20-75 |
| Cause of injury, no. (%) |  |  |
| Transportation accidents | 26 (47.3) | 70 (57.9) |
| Falls | 18 (32.7) | 35 (28.9) |
| Other | 11 (20.0) | 16 (13.2) |
| GCS^b^ | 15 [15~15] | 15 [15~15] |
| GOSE^b^ | 7 [6~8] | 7 [6~8] |
| BAI^b^ | 4.5 [2~9] | 7 [2.25~12] |
| BDI^b^ | 6 [2~10.5] | 7 [2~12] |
| DHI^b^ | 12 [2~31] | 26 [10.5~42] |
| PSQI^b^ | 6 [5~8.75] | 7 [5~9] |
| Serum IGF-1 (ng/mL)^a^ | 144.75 ± 64.63 | 175.30± 80.74 |
| ^a^mean ± standard deviation. ^b^median [interquartile range]. *GCS* Glasgow Coma Scale, *GOSE* Extended Glasgow Outcome Scale, *BAI* Beck Anxiety Inventory, *BDI* Beck Depression Inventory, *DHI* Dizziness Handicap Inventory, *PSQI* Pittsburgh Sleep Quality Index, *IGF-1* Insulin-like growth factor 1 | | |

| **Table S3. Sex-stratified analyses for Beck Anxiety Inventory (BAI) score** | | | | | | | |
| --- | --- | --- | --- | --- | --- | --- | --- |
| **Sex** | **SNP** | **Genotype** | **BAI score** | | **OR (95% CI)** | **Additive model^a^** | |
|  |  |  | **BAI ≤7** | **BA I>7** |  | ***p-*value** | **Bonferroni** |
| **Males** | rs35767 | GG | 15 (39.5) | 5 (31.2) | 1.38 (0.58-3.29) | 0.468 | 1.000 |
|  |  | GA | 18 (47.4) | 8 (50.0) |  |  |  |
|  |  | AA | 5 (13.2) | 3 (18.8) |  |  |  |
|  | rs5742612 | AA | 16 (42.1) | 4 (26.7) | 1.96 (0.75-5.06) | 0.160 | 1.000 |
|  |  | AG | 19 (50.0) | 8 (53.3) |  |  |  |
|  |  | GG | 3 (7.9) | 3 (20.0) |  |  |  |
|  | rs7136446 | TT | 24 (63.2) | 10 (62.5) | 1.10 (0.39-3.12) | 0.853 | 1.000 |
|  |  | TC | 13 (34.2) | 5 (31.2) |  |  |  |
|  |  | CC | 1 (2.6) | 1 (6.2) |  |  |  |
|  | rs972936 | CC | 12 (31.6) | 2 (12.5) | 1.79 (0.74-4.33) | 0.187 | 1.000 |
|  |  | CT | 18 (47.4) | 9 (56.2) |  |  |  |
|  |  | TT | 8 (21.1) | 5 (31.2) |  |  |  |
|  | rs2072592 | CC | 19 (51.4) | 7 (43.8) | 1.57 (0.63-3.86) | 0.331 | 1.000 |
|  |  | CT | 16 (43.2) | 6 (37.5) |  |  |  |
|  |  | TT | 2 (5.4) | 3 (18.8) |  |  |  |
| **Females** | rs35767 | GG | 29 (46.0) | 23 (42.6) | 1.04 (0.61-1.78) | 0.892 | 1.000 |
|  |  | GA | 26 (41.3) | 25 (46.3) |  |  |  |
|  |  | AA | 8 (12.7) | 6 (11.1) |  |  |  |
|  | rs5742612 | AA | 31 (48.4) | 24 (45.3) | 1.04 (0.60-1.80) | 0.890 | 1.000 |
|  |  | AG | 26 (40.6) | 24 (45.3) |  |  |  |
|  |  | GG | 7 (10.9) | 5 (9.4) |  |  |  |
|  | rs7136446 | TT | 43 (67.2) | 27 (50.0) | 1.61 (0.89-2.93) | 0.112 | 1.000 |
|  |  | TC | 17 (26.6) | 23 (42.6) |  |  |  |
|  |  | CC | 4 (6.2) | 4 (7.4) |  |  |  |
|  | rs972936 | CC | 13 (20.6) | 17 (31.5) | 0.68 (0.41-1.15) | 0.152 | 1.000 |
|  |  | CT | 32 (50.8) | 26 (48.1) |  |  |  |
|  |  | TT | 18 (28.6) | 11 (20.4) |  |  |  |
|  | rs2072592 | CC | 33 (52.4) | 27 (50.9) | 1.07 (0.59-1.93) | 0.820 | 1.000 |
|  |  | CT | 26 (41.3) | 22 (41.5) |  |  |  |
|  |  | TT | 4 (6.3) | 4 (7.5) |  |  |  |
| ^a^Adjusted for age. *OR* odds ratio, *CI* confidence interval | | | | | | | |

| **Table S4. Sex-stratified analyses for Beck Depression Inventory (BDI) score** | | | | | | | |
| --- | --- | --- | --- | --- | --- | --- | --- |
| **Sex** | **SNP** | **Genotype** | **BDI score** | | **OR (95% CI)** | **Additive model^a^** | |
|  |  |  | **BDI ≤9** | **BDI >9** |  | ***p-*value** | **Bonferroni** |
| **Males** | rs35767 | GG | 16 (41.0) | 5 (31.2) | 1.73 (0.72-4.16) | 0.212 | 1.000 |
|  |  | GA | 19 (48.7) | 7 (43.8) |  |  |  |
|  |  | AA | 4 (10.3) | 4 (25.0) |  |  |  |
|  | rs5742612 | AA | 16 (42.1) | 5 (31.2) | 2.13 (0.83-5.46) | 0.107 | 1.000 |
|  |  | AG | 20 (52.6) | 7 (43.8) |  |  |  |
|  |  | GG | 2 (5.3) | 4 (25.0) |  |  |  |
|  | rs7136446 | TT | 23 (59.0) | 12 (75) | 0.45 (0.13-1.51) | 0.169 | 1.000 |
|  |  | TC | 14 (35.9) | 4 (25) |  |  |  |
|  |  | CC | 2 (5.1) | 0 (0) |  |  |  |
|  | rs972936 | CC | 10 (25.6) | 5 (31.2) | 1.08 (0.47-2.50) | 0.855 | 1.000 |
|  |  | CT | 21 (53.8) | 6 (37.5) |  |  |  |
|  |  | TT | 8 (20.5) | 5 (31.2) |  |  |  |
|  | rs2072592 | CC | 19 (48.7) | 8 (53.3) | 1.57 (0.63-3.88) | 0.333 | 1.000 |
|  |  | CT | 19 (48.7) | 3 (20.0) |  |  |  |
|  |  | TT | 1 (2.6) | 4 (26.7) |  |  |  |
| **Females** | rs35767 | GG | 34 (44.2) | 18 (45) | 0.92 (0.52-1.63) | 0.778 | 1.000 |
|  |  | GA | 33 (42.9) | 18 (45) |  |  |  |
|  |  | AA | 10 (13.0) | 4 (10) |  |  |  |
|  | rs5742612 | AA | 35 (44.9) | 20 (51.3) | 0.80 (0.44-1.44) | 0.448 | 1.000 |
|  |  | AG | 34 (43.6) | 16 (41.0) |  |  |  |
|  |  | GG | 9 (11.5) | 3 (7.7) |  |  |  |
|  | rs7136446 | TT | 50 (64.1) | 20 (50.0) | 1.49 (0.81-2.73) | 0.201 | 1.000 |
|  |  | TC | 23 (29.5) | 17 (42.5) |  |  |  |
|  |  | CC | 5 (6.4) | 3 (7.5) |  |  |  |
|  | rs972936 | CC | 19 (24.7) | 11 (27.5) | 1.10 (0.64-1.89) | 0.723 | 1.000 |
|  |  | CT | 41 (53.2) | 17 (42.5) |  |  |  |
|  |  | TT | 17 (22.1) | 12 (30.0) |  |  |  |
|  | rs2072592 | CC | 35 (45.5) | 25 (64.1) | 0.62 (0.32-1.20) | 0.144 | 1.000 |
|  |  | CT | 37 (48.1) | 11 (28.2) |  |  |  |
|  |  | TT | 5 (6.5) | 3 (7.7) |  |  |  |
| ^a^Adjusted for age. *OR* odds ratio, *CI* confidence interval | | | | | | | |

| **Table S5. Sex-stratified analyses for Dizziness Handicap Inventory (DHI) score** | | | | | | | |
| --- | --- | --- | --- | --- | --- | --- | --- |
| **Sex** | **SNP** | **Genotype** | **DHI score** | | **OR (95% CI)** | **Additive model^a^** | |
|  |  |  | **DHI ≤30** | **DHI >30** |  | ***p-*value** | **Bonferroni** |
| **Males** | rs35767 | GG | 17 (42.5) | 3 (21.4) | 1.87 (0.75-4.64) | 0.169 | 1.000 |
|  |  | GA | 18 (45.0) | 8 (57.1) |  |  |  |
|  |  | AA | 5 (12.5) | 3 (21.4) |  |  |  |
|  | rs5742612 | AA | 18 (45) | 3 (21.4) | 1.91 (0.74-4.93) | 0.176 | 1.000 |
|  |  | AG | 18 (45) | 9 (64.3) |  |  |  |
|  |  | GG | 4 (10) | 2 (14.3) |  |  |  |
|  | rs7136446 | TT | 28 (70.0) | 7 (50.0) | 2.18 (0.75-6.31) | 0.150 | 1.000 |
|  |  | TC | 11 (27.5) | 6 (42.9) |  |  |  |
|  |  | CC | 1 (2.5) | 1 (7.1) |  |  |  |
|  | rs972936 | CC | 13 (32.5) | 2 (14.3) | 2.58 (1.02-6.55) | 0.037* | 0.740 |
|  |  | CT | 20 (50.0) | 6 (42.9) |  |  |  |
|  |  | TT | 7 (17.5) | 6 (42.9) |  |  |  |
|  | rs2072592 | CC | 21 (53.8) | 5 (35.7) | 1.46 (0.58-3.71) | 0.424 | 1.000 |
|  |  | CT | 14 (35.9) | 8 (57.1) |  |  |  |
|  |  | TT | 4 (10.3) | 1 (7.1) |  |  |  |
| **Females** | rs35767 | GG | 30 (42.3) | 23 (50.0) | 0.65 (0.36-1.17) | 0.146 | 1.000 |
|  |  | GA | 31 (43.7) | 21 (45.7) |  |  |  |
|  |  | AA | 10 (14.1) | 2 (4.3) |  |  |  |
|  | rs5742612 | AA | 33 (46.5) | 23 (50.0) | 0.68 (0.38-1.23) | 0.195 | 1.000 |
|  |  | AG | 28 (39.4) | 22 (47.8) |  |  |  |
|  |  | GG | 10 (14.1) | 1 (2.2) |  |  |  |
|  | rs7136446 | TT | 49 (69.0) | 21 (44.7) | 2.73 (1.44-5.18) | 0.001** | 0.020* |
|  |  | TC | 21 (29.6) | 19 (40.4) |  |  |  |
|  |  | CC | 1 (1.4) | 7 (14.9) |  |  |  |
|  | rs972936 | CC | 13 (18.3) | 16 (34.8) | 0.60 (0.35-1.04) | 0.062 | 1.000 |
|  |  | CT | 38 (53.5) | 21 (45.7) |  |  |  |
|  |  | TT | 20 (28.2) | 9 (19.6) |  |  |  |
|  | rs2072592 | CC | 34 (48.6) | 26 (56.5) | 0.74 (0.40-1.39) | 0.344 | 1.000 |
|  |  | CT | 31 (44.3) | 18 (39.1) |  |  |  |
|  |  | TT | 5 (7.1) | 2 (4.3) |  |  |  |
| ^a^Adjusted for age. *indicates *p*<0.05, **indicates *p*<0.01. *OR* odds ratio, *CI* confidence interval | | | | | | | |

| **Table S6. Sex-stratified analyses for Pittsburgh Sleep Quality Index (PSQI) score** | | | | | | | |
| --- | --- | --- | --- | --- | --- | --- | --- |
| **Sex** | **SNP** | **Genotype** | **PSQI score** | | **OR (95% CI)** | **Additive model^a^** | |
|  |  |  | **PSQI ≤8** | **PSQI >8** |  | ***p-*value** | **Bonferroni** |
| **Males** | rs35767 | GG | 12 (32.4) | 7 (53.8) | 0.67 (0.24-1.81) | 0.421 | 1.000 |
|  |  | GA | 21 (56.8) | 4 (30.8) |  |  |  |
|  |  | AA | 4 (10.8) | 2 (15.4) |  |  |  |
|  | rs5742612 | AA | 13 (35.1) | 6 (50.0) | 0.84 (0.30-2.36) | 0.744 | 1.000 |
|  |  | AG | 21 (56.8) | 4 (33.3) |  |  |  |
|  |  | GG | 3 (8.1) | 2 (16.7) |  |  |  |
|  | rs7136446 | TT | 22 (59.5) | 9 (69.2) | 0.59 (0.17-2.03) | 0.388 | 1.000 |
|  |  | TC | 13 (35.1) | 4 (30.8) |  |  |  |
|  |  | CC | 2 (5.4) | 0 (0) |  |  |  |
|  | rs972936 | CC | 8 (21.6) | 5 (38.5) | 0.71 (0.27-1.85) | 0.475 | 1.000 |
|  |  | CT | 21 (56.8) | 5 (38.5) |  |  |  |
|  |  | TT | 8 (21.6) | 3 (23.1) |  |  |  |
|  | rs2072592 | CC | 17 (47.2) | 8 (61.5) | 0.89 (0.32-2.45) | 0.815 | 1.000 |
|  |  | CT | 17 (47.2) | 3 (23.1) |  |  |  |
|  |  | TT | 2 (5.6) | 2 (15.4) |  |  |  |
| **Females** | rs35767 | GG | 32 (44.4) | 17 (47.2) | 0.93 (0.51-1.70) | 0.814 | 1.000 |
|  |  | GA | 32 (44.4) | 15 (41.7) |  |  |  |
|  |  | AA | 8 (11.1) | 4 (11.1) |  |  |  |
|  | rs5742612 | AA | 35 (48.6) | 17 (47.2) | 0.98 (0.53-1.83) | 0.958 | 1.000 |
|  |  | AG | 30 (41.7) | 16 (44.4) |  |  |  |
|  |  | GG | 7 (9.7) | 3 (8.3) |  |  |  |
|  | rs7136446 | TT | 48 (66.7) | 16 (43.2) | 1.97 (1.04-3.74) | 0.035* | 0.700 |
|  |  | TC | 20 (27.8) | 17 (45.9) |  |  |  |
|  |  | CC | 4 (5.6) | 4 (10.8) |  |  |  |
|  | rs972936 | CC | 14 (19.4) | 13 (36.1) | 0.57 (0.31-1.03) | 0.058 | 1.000 |
|  |  | CT | 38 (52.8) | 17 (47.2) |  |  |  |
|  |  | TT | 20 (27.8) | 6 (16.7) |  |  |  |
|  | rs2072592 | CC | 36 (50.7) | 19 (52.8) | 0.96 (0.49-1.88) | 0.896 | 1.000 |
|  |  | CT | 31 (43.7) | 15 (41.7) |  |  |  |
|  |  | TT | 4 (5.6) | 2 (5.6) |  |  |  |
| ^a^Adjusted for age. *indicates *p*<0.05. *OR* odds ratio, *CI* confidence interval | | | | | | | |

| **Table S7. Single nucleotide polymorphisms (SNPs)-sex interaction analyses for neuropsychiatric symptoms following mild traumatic brain injury (mTBI)** | | | | | | | | | | | |  |
| --- | --- | --- | --- | --- | --- | --- | --- | --- | --- | --- | --- | --- |
| **SNP** | **BAI** | |  | **BDI** | |  | **DHI** | |  | **PSQI** | | |
|  | **OR (95% CI)** | ***p-*value^a^** |  | **OR (95% CI)** | ***p-*value^a^** |  | **OR (95% CI)** | ***p-*value^a^** |  | **OR (95% CI)** | ***p-*value^a^** | |
| rs35767 | 0.95 (0.75-1.20) | 0.665 |  | 0.88 (0.71-1.10) | 0.261 |  | 0.80 (0.64-1.01) | 0.059 |  | 1.07 (0.85-1.36) | 0.563 | |
| rs5742612 | 0.88 (0.69-1.13) | 0.322 |  | 0.82 (0.65-1.03) | 0.088 |  | 0.81 (0.64-1.02) | 0.076 |  | 1.03 (0.81-1.32) | 0.787 | |
| rs7136446 | 1.10 (0.83-1.44) | 0.506 |  | 1.27 (0.97-1.65) | 0.079 |  | 1.08 (0.83-1.40) | 0.561 |  | 1.27 (0.97-1.65) | 0.080 | |
| rs972936 | 0.97 (0.78-1.21) | 0.794 |  | 0.96 (0.78-1.19) | 0.724 |  | 0.95 (0.77-1.18) | 0.636 |  | 1.19 (0.95-1.49) | 0.128 | |
| rs2072592 | 0.92 (0.72-1.18) | 0.510 |  | 0.83 (0.65-1.05) | 0.117 |  | 0.86 (0.68-1.11) | 0.248 |  | 1.00 (0.78- 1.29) | 0.982 | |
| ^a^The *p*-value for interaction term was adjusted for age under additive model. *BAI* Beck Anxiety Inventory, *BDI* Beck Depression Inventory, *DHI* Dizziness Handicap Inventory, *PSQI* Pittsburgh Sleep Quality Index, *OR* odds ratio, *CI* confidence interval | | | | | | | | | | | |  |

| **Table S8. Expression quantitative trail loci (eQTL) results from Genotype-tissue expression (GTEx)** | | | | | |
| --- | --- | --- | --- | --- | --- |
| **SNP** | **Gene symbol** | ***p-*value** | **NES** | **Tissue** | **Action^a^** |
| **rs7136446** | *WASHC3* | 4.6E-9 | 0.25 | Esophagus-Mucosa | CC<CT<TT |
|  | *WASHC3* | 2.3E-6 | 0.09 | Whole Blood | CC<CT<TT |
|  | *WASHC3* | 8.7E-5 | 0.13 | Skin-Sun Exposed (Lower leg) | CC<CT<TT |
|  | *RP11-210L7.3* | 7.2E-5 | 0.19 | Testis | CC<CT<TT |
|  | *HELLPAR* | 4.4E-5 | 0.11 | Muscle-Skeletal | CT<CC<TT |
|  |  |  |  |  |  |
| **rs972936** | *PARPBP* | 1.1E-4 | -0.20 | Testis | TT>TC>CC |
|  | *PARPBP* | 1.3E-6 | -0.23 | Adipose-Subcutaneous | TT>TC>CC |
|  | *PARPBP* | 1.4E-6 | -0.10 | Cells-Cultured fibroblasts | TT>TC>CC |
|  | *RP11-210L7.3* | 7.5E-14 | 0.40 | Testis | TT<TC<CC |
|  | *WASHC3* | 5.7E-26 | 0.47 | Esophagus-Mucosa | TT<TC<CC |
|  | *WASHC3* | 1.5E-15 | 0.32 | Skin-Not Sun Exposed (Suprapubic) | TT<TC<CC |
|  | *WASHC3* | 1.0E-14 | 0.27 | Skin-Sun Exposed (Lower leg) | TT<TC<CC |
|  | *WASHC3* | 3.6E-10 | 0.20 | Nerve-Tibial | TT<TC<CC |
|  | *WASHC3* | 1.5E-10 | 0.26 | Heart-Left Ventricle | TT<TC<CC |
|  | *WASHC3* | 9.4E-12 | 0.14 | Whole Blood | TT<TC<CC |
|  | *WASHC3* | 3.0E-10 | 0.18 | Muscle-Skeletal | TT<TC<CC |
|  | *WASHC3* | 6.6E-9 | 0.17 | Breast-Mammary Tissue | TT<TC<CC |
|  | *WASHC3* | 1.2E-8 | 0.20 | Colon-Transverse | TT<TC<CC |
|  | *WASHC3* | 8.6E-8 | 0.15 | Esophagus-Muscularis | TT<TC<CC |
|  | *WASHC3* | 7.1E-9 | 0.31 | Stomach | TT<TC<CC |
|  | *WASHC3* | 1.3E-7 | 0.21 | Adipose-Visceral (Omentum) | TT<TC<CC |
|  | *WASHC3* | 8.9E-7 | 0.18 | Adipose-Subcutaneous | TT<TC<CC |
|  | *WASHC3* | 1.0E-7 | 0.14 | Artery-Tibial | TT<TC<CC |
|  | *WASHC3* | 1.0E-6 | 0.25 | Heart-Atrial Appendage | TT<TC<CC |
|  | *WASHC3* | 1.3E-6 | 0.27 | Adrenal Gland | TT<TC<CC |
|  | *WASHC3* | 1.1E-7 | 0.34 | Pancreas | TT<TC<CC |
|  | *WASHC3* | 1.0E-8 | 0.18 | Thyroid | TT<TC<CC |
|  | *WASHC3* | 1.4E-6 | 0.20 | Artery-Aorta | TT<TC<CC |
|  | *WASHC3* | 4.4E-6 | 0.16 | Cells-Cultured fibroblasts | TT<TC<CC |
|  | *WASHC3* | 4.6E-6 | 0.12 | Lung | TT<TC<CC |
|  | *WASHC3* | 2.4E-5 | 0.24 | Spleen | TT<TC<CC |
| ^a^The median expression level of each genotype. *NES* normalized effect size, *WASHC3* WASH complex subunit 3, *RP11-210L7.3* long intergenic non-protein coding RNA 2456, *HELLPAR* HELLP associated long non-coding RNA, *PARPBP* PARP1 binding protein. Data Source: GTEx Analysis Release V8. | | | | | |

| **Table S9. Functional annotation by HaploReg V4.1 and RegulomeDB** | | | | | | | | | | |
| --- | --- | --- | --- | --- | --- | --- | --- | --- | --- | --- |
| **SNP** | **Risk allele** | **dbSNP** |  | **HaploReg V4.1** | | | | |  | **RegulomeDB** |
|  |  |  |  | **Promoter**  **histone marks** | **Enhancer**  **histone marks** | **DNAse** | **Proteins**  **bound** | **Motifs**  **changed** |  |  |
| **rs7136446** | C | intronic |  | ― | ― | ― | ― | HDAC2, NRSF, Sin3Ak-20 |  | TF binding +  DNase peak |
| **rs972936** | T | intronic |  | ― | 4 tissues | BLD | ― | NF-κB, STAT |  | TF binding or  DNase peak |
| *HDAC2* histone deacetylase 2, *NRSF* neuron-restrictive silencer factor, *NF-κB* nuclear factor-κB, *STAT* signal transducer and activator of transcription, *TF* transcription factor | | | | | | | | | | |

| **Table S10.** **Expression quantitative trait loci (eQTL) results of rs7136446 and rs972936 with insulin-like growth factor 1 *(IGF-1*) expressions in brain tissues from Genotype-tissue expression (GTEx)** | | | | | | |
| --- | --- | --- | --- | --- | --- | --- |
| **Gene** | **SNP** | **Risk allele** | **Brain tissue** | ***p*-value** | **NES** | **Action^a^** |
| *IGF-1* | rs7136446 | C | Brain_Hippocampus | 0.013 | 0.21 | CC<CT<TT |
|  | rs972936 | T | Brain-Frontal Cortex (BA9) | 0.036 | 0.20 | TT<TC<CC |
|  | rs972936 | T | Brain_Hippocampus | 0.047 | 0.22 | TT<TC<CC |
| ^a^The median expression level of each genotype. *NES* mormalized effect size. Data Source: GTEx Analysis Release V8. | | | | | | |

| **Table S11.** **Association between insulin-like growth factor 1 (*IGF-1*) expression levels and Alzheimer’s disease (AD) in brain donors with no history of traumatic brain injury (TBI)** | | | | | | | | |
| --- | --- | --- | --- | --- | --- | --- | --- | --- |
| **Brain region** | **Alzheimer’s disease** | | |  | **No Dementia** | | | ***p*-value** |
|  | **Samples** | **Mean** | **SD** |  | **Samples** | **Mean** | **SD** |  |
| Parietal white matter (FWM) | 15 | 0.883 | 0.310 |  | 27 | 0.752 | 0.246 | 0.174 |
| Parietal cortex (PCx) | 15 | 1.012 | 0.239 |  | 27 | 1.005 | 0.181 | 0.926 |
| Temporal cortex (TCx) | 15 | 1.078 | 0.225 |  | 28 | 1.043 | 0.205 | 0.623 |
| Hippocampus (HIP) | 13 | 1.343 | 0.338 |  | 29 | 1.366 | 0.284 | 0.830 |
| *SD* standard deviation. | | | | | | | | |
